# Discriminative Method for Crack Detection Signals in Balanced-Field Electromagnetic Technique Based on Amplitude-Phase Composite Figure

**DOI:** 10.3390/s22187000

**Published:** 2022-09-15

**Authors:** Jiayin Li, Lijian Yang, Wenxue Zheng, Bin Liu

**Affiliations:** School of Information Science and Engineering, Shenyang University of Technology, Shenyang 110870, China

**Keywords:** amplitude-phase composite figure, balanced-field electromagnetic technique, sensor tilt jitter, differentiation of detection signals, phase noise

## Abstract

The balanced-field electromagnetic technique is an effective in-line inspection method for pipeline cracks. To address the problem that the interference signal generated by the tilt jitter of the sensor during the detection process affects the judgment of cracks, this paper proposes a method to differentiate the crack detection signal from the sensor jitter signal by using an amplitude-phase composite figure. The generation principle of the detection signal was analyzed by using the mutual inductance model, and the amplitude-phase composite figure was constructed by using the components of the detection signal after quadrature demodulation. The feasibility of using the phase as a signal discrimination feature was illustrated by finite element simulations, and the characteristics of the amplitude-phase composite figure were determined. The validity of the proposed method was verified experimentally. The results show that the crack detection signal and the signal generated by the sensor jitter are of the same frequency with similar waveforms and significantly different phases. The phase base value of the crack detection signal ranges from 35° to 55°, and the phase base value of the jitter signal is −4°. In terms of the characteristics of the amplitude-phase composite figure, the crack detection signal distribution is symmetrical about the origin in the first and third quadrants, and the axial crack is closer to the *Y*-axis than the circumferential crack; the jitter signal is distributed in the second and fourth quadrants and has a very small angle to the *X*-axis. In addition, the proposed method effectively weakens the observation of the phase noise region in the detection signal of the balanced-field electromagnetic technique.

## 1. Introduction

Oil and gas pipelines have become the main way to transport energy with their economic and efficient characteristics. The safe operation of pipelines ensures the effective transportation and full utilization of oil and gas energy [1]. However, due to the special service environment of buried pipelines, it is very easy to cause catastrophic accidents due to corrosion, external loads, and other factors that lead to thinning of the pipe wall or defects. Among them, crack defects seriously affect the pressure-bearing capacity and safety performance of oil and gas pipelines as the most important failure factor [2,3]. Therefore, the effective detection of pipeline cracks is of great significance to extend the life of pipelines and ensure pipeline safety.

Pipeline crack detection methods include off-line detection and in-line detection [4]. However, long-distance oil and gas pipelines have the characteristics of long laying distance and are buried underground, and the location of cracks is also random. Therefore, it is not applicable to conduct the off-line detection of excavating the whole line of buried pipelines. Usually, off-line detection is used in excavation verification of in-line detection results [5]. In-line inspection (ILI) technology is recognized as the most effective means of detecting defects in long-distance oil and gas pipelines, and it is an in-line detection method that does not affect the in-service operation of the pipeline. The pipeline inspection gauge (PIG) is the main implementation method of ILI technology [6,7]. PIG is used to complete the inspection inside the pipeline without affecting the normal operation of the pipeline by carrying non-destructive testing sensors, signal processing systems, acquisition and storage systems, and other devices. In addition, it uses the pressure difference of the transport medium inside the pipeline as the driving force for travel [8,9]. The detection techniques currently used in the field of ILI have certain limitations due to cracks having small openings, random shapes, and varying directions. For example, the most mainstream magnetic flux leakage detection (MFL) technology in ILI has good results in detecting defects with a certain volume [10]. However, it is difficult to detect cracks with MFL technology [11]. Ultrasonic testing (UT) is also a relatively mature crack detection method applied to the PIG, but it is not suitable for pipelines with non-liquid media due to the limitations of the coupling agent [12,13]. Electromagnetic acoustic transducer (EMAT) techniques, although not requiring a coupling agent, can only detect cracks in a specific direction, and the detection speed is generally limited to 2.5 m/s [14]. In addition to these techniques, which have been used in the field of ILI, there are a number of techniques that have good detection results for crack defects. For example, Wilson and Tian have proposed a technique that combined PMFL and PMR that enables the detection of surface as well as buried defects [15]. However, this technique uses very low frequencies so that the power consumption is very high. The power supply of PIG is limited, so it is more suitable for off-line detection. Machine vision technology is also widely used for the detection of defects such as cracks and deformations, with good detection accuracy [16,17]. However, the visual inspection of full excavation of buried pipelines is also inappropriate. There are also some composite detection techniques in the field of pipeline defect detection. For example, Wang et al. proposed a combined AC and DC magnetization technique that avoids the problem of insufficient sensitivity of MFL detection under DC magnetization [18]. Liu et al. proposed circumferential and axial magnetization to achieve the detection of volume defects in any direction separately [19]. However, the application of these methods to the PIG has the problem of complex structure, and the complex structure also poses a challenge to the passing performance of the PIG. The balanced-field electromagnetic technique is an emerging electromagnetic non-destructive testing technology. First proposed by TESTEX (USA), an off-line detection system that works at a speed below 0.3 m/s was developed with the balanced-field electromagnetic technique for pipeline cracks. Beyond this, there has been no published research on the balanced field electromagnetic technique. However, this detection system is not suitable for the application environment of long oil and gas pipelines due to the extremely low speed and the offline detection form. The authors have previously demonstrated the applicability of the balanced-field electromagnetic technique for crack detection in any direction and developed a highly sensitive sensor and a PIG for the in-line inspection of cracks in long-distance oil and gas pipelines [20,21]. Because the balanced-field electromagnetic technique has the characteristics of high sensitivity to small opening defects and the ability to detect in any direction with a single structure, it has good applicability and great development potential for in-line crack detection of long-distance oil and gas pipelines. However, there will be sludge, impurities, and other attachments in the pipeline due to incomplete pigging. During the in-line inspection process, the balanced-field electromagnetic technique sensor, which is close to the pipeline wall, experiences tilt jitter to varying degrees when the PIG passes through these positions [22]. The resulting interference signals greatly affect the judgment of crack defects. Therefore, the study of the discrimination method of the balanced-field electromagnetic technique crack detection signal, particularly from jitter signals, can avoid misjudgment and missed detection in the analysis of the detection data. Accurate identification provides a technical basis for the evaluation of pipeline defects.

At present, there is less research on the balanced-field electromagnetic technique, and there is no published research content on the detection signal of the balanced-field electromagnetic technique. The more common crack identification method is to use neural networks and other methods to automatically identify the detected image or the waveform characteristics of the signal [23,24,25]. For example, Tang et al. proposed a U-net neural network algorithm and an improved image refinement algorithm to achieve accurate visual recognition and width measurement of cracks [26]. However, in the balanced-field electromagnetic technique, the jitter signal has similar waveform characteristics to the crack detection signal, so it is not possible to distinguish between the two utilizing waveform recognition. In addition, there is the signal generated by sensor jitter, as a kind of interference signal; the interference effect of the jitter signal can also be reduced by increasing the signal-to-noise ratio [27]. For alternating current detection techniques, the most commonly used denoising method is the lock-in amplifier, which is very effective in suppressing noise that is not at the same frequency as the detection signal [28,29]. However, the signal generated by the jitter is at the same frequency as the crack detection signal, so it is difficult to filter out the jitter signal using conventional frequency domain denoising or to distinguish the crack from the jitter generated signal using the difference in frequency. Jitter signals have also been investigated in other pipeline in-line inspection techniques. For example, there is a sensor jitter perpendicular to the detection plane in eddy current testing. Lu et al. proposed an index to compensate for the peak frequency and developed an algorithm to compensate for the jitter effect using this index [30]. Fu et al. shifted the trajectory of each harmonic in the impedance plane after the fast Fourier transform of the pulsed eddy current detection signal and reconstructed the signal by inverse fast Fourier transform to eliminate the interference of vertical jitter [31]. Similar jitter interference exists in the magnetic flux leakage technique, which affects the amplitude judgment of defect signals [32]. Jia et al. proposed a processing method using the correlation between adjacent sensor signals, which effectively suppresses this jitter noise [33]. In addition, the alternating current field measurement (ACFM) technique, which is well used in crack detection, also has related research on the jitter interference processing in the vertical direction. Yuan et al. demonstrated the insensitivity of the Bz signal to jitter effects in ACFM and proposed a signal gradient algorithm to achieve crack identification on the weld and in the heat-affected zone [34]. These studies on jitter interference signal processing are all directed at jitter in the vertical direction of the sensor. However, the self-zero characteristic of the balanced-field electromagnetic technique does not produce distorted signals when vertical jitter occurs in the sensor [21]. Jitter in the tilt of the sensor produces a signal with a waveform similar to that of the crack detection signal. The above indicates that methods that are effective in other areas for identifying cracks or eliminating jitter are not applicable to the detection signals of balanced-field electromagnetic technique because cracks and jitter signals have similar waveforms and the same frequency. There is currently no method that can effectively differentiate between crack detection signals and jitter signals of the balanced-field electromagnetic technique. Therefore, further research should be conducted on the identification method of balanced-field electromagnetic technique crack detection signals to make the balanced-field electromagnetic technique more perfect in practice, especially the differentiation from jitter signals.

This paper proposes a discriminate method for crack detection signals of the balanced-field electromagnetic technique by using the amplitude-phase composite figure, which can characterize both the amplitude and the phase of the detection signal. The aim is to provide a practically feasible method for the differentiation of cracks from jitter in balanced-field electromagnetic technique detection signals. Firstly, the generation principle of the crack detection signal and the jitter signal of the balanced-field electromagnetic technique is analyzed, and the co-frequency of the two is illustrated. Secondly, orthogonal demodulation is used to separate the detection signal into two mutually orthogonal components, and this is used as the parametric equation to construct the amplitude-phase composite figure that characterizes both the signal amplitude and phase. The amplitude and phase variations of the crack detection signal and the jitter signal are analyzed using finite element simulations, and the characteristics of the amplitude-phase composite figure of the crack and sensor jitter signal are determined. Finally, the validity of the proposed discrimination method and the correctness of the theoretical analysis are verified through experiments, and the engineering applicability of the method is demonstrated through practical pipeline pulling experiments.

## 2. Theoretical Analysis and Methods

### 2.1. Crack Detection Principle and Jitter Signal Analysis

**Figure 1:**
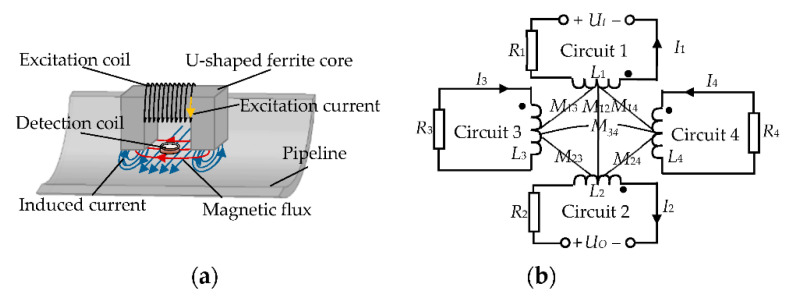
The detection principle of balanced-field electromagnetic technique: (**a**) Schematic diagram of the electromagnetic distribution on the surface of the pipeline; (**b**) Mutual inductance model for the detection of balanced-field electromagnetic technique.

To achieve high sensitivity detection for pipeline cracks, the balanced-field electromagnetic technique sensor uses a pair of spatially orthogonal and symmetrically arranged coils for excitation and detection, generating the alternating magnetic field and eddy current field through the U-shaped ferrite magnetic conductivity to the surface of the pipeline [35]. Compared to other methods which add another magnetization direction to the structure, the balanced-field electromagnetic technique uses two fields generated by one structure at the same time to detect cracks in any direction, which is one of the more obvious advantages of the balanced-field electromagnetic technique in the field of in-line inspection in long-distance oil and gas pipelines. The core detection principle of the balanced-field electromagnetic technique is the use of the detection coil to pick up electromagnetic field distortions on the surface of the pipeline. When the pipeline is free of defects, the magnetic flux directly below the excitation coil is distributed parallel to the pipeline surface on the one hand, and the orthogonal structure makes the magnetic field generated by the excitation current not to cause the detection coil to produce an induced voltage [20]. On the other hand, the changing magnetic field produces the induced current that is symmetrical about the detection coil on the pipeline surface, and the induced magnetic field from the induced current does not pass perpendicular to the detection coil. This forms an electromagnetic balance state, as shown in Figure 1a. The pipeline cracks will break this electromagnetic balance state due to discontinuities in magnetic or electrical permeability. The sensor’s detection coil will pick up the interaction between the crack and the pipeline, resulting in the induced voltage [21]. To further analyze the process of generating detection signals from this interaction, a mutual inductance model is established by impedance analysis. The balanced-field electromagnetic technique sensor and the pipeline directly below the sensor are equated as a series connection of resistance and inductance, forming an excitation coil circuit (circuit 1), a detection coil circuit (circuit 2), and the pipeline circuits below the two legs of the U-shaped ferrite (circuits 3 and 4), respectively, as shown in Figure 1b.

According to the equivalence relation in Figure 1b, the mutual inductance model for the balanced-field electromagnetic technique is expressed as:

(1) $$\left[\begin{array}{cccc} Z_{1} & j\omega M_{12} & j\omega M_{13} & j\omega M_{14}\\ j\omega M_{21} & Z_{2} & j\omega M_{23} & j\omega M_{24}\\ j\omega M_{31} & j\omega M_{32} & Z_{3} & j\omega M_{34}\\ j\omega M_{41} & j\omega M_{42} & j\omega M_{43} & Z_{4} \end{array}\right]\left[\begin{array}{c} I_{1}\\ I_{2}\\ I_{3}\\ I_{4} \end{array}\right]=\left[\begin{array}{c} U_{I}\\ U_{O}\\ 0\\ 0 \end{array}\right]$$

where *M_ij_* is the mutual inductance coefficient between the circuits. *I_i_* is the current of each circuit. *Z_i_* is the complex impedance of the circuit, which is equal to the sum of the resistance *R_i_* and the inductive reactance $j\omega L_{i}$. *i* and *j* both are 1, 2, 3, and 4. Due to the self-zero characteristic of the balanced-field electromagnetic technique, the mutual inductance coefficients *M*_12_ and *M*_21_ between the excitation and detection coils are both zero [21]. The excitation coil voltage *U_I_* and the detection coil induced voltage *U_O_*, respectively, are obtained as

(2) $$U_{I}=Z_{1}I_{1}+j\omega M_{13}I_{3}+j\omega M_{14}I_{4}$$

(3) $$U_{O}=Z_{2}I_{2}+j\omega M_{23}I_{3}+j\omega M_{24}I_{4}$$

Equations (2) and (3) show that the detection voltage of the balanced-field electromagnetic technique is generated by the change in mutual inductance between the pipeline and the detection coil circuit so that the detection signal is at the same frequency as the excitation signal. According to Neumann’s formula [36], the mutual inductance coefficients *M*_23_ and *M*_24_ between the detection coil circuit and the pipeline circuit can be expressed as:

(4) $$M_{2i}=\frac{\mu_{0}N_{2}}{4\pi }\oint_{l_{2}}\oint_{l_{i}}\frac{dl_{2}dl_{i}}{r_{2i}}$$

where *N*_2_ is the number of turns of the detection coil; ***l***_2_ and ***l***_*i*_ are the equivalent current elements of the single-turn detection coil and the equivalent current elements of the pipeline surface, respectively; ***r***_2*i*_ is the distance between each of the pipeline on the left and right sides of the detection coil and the detection coil.

When the pipeline is free of defects, the current *I*_3_ in circuit 3 is equal in size and opposite in direction to the current *I*_4_ in circuit 4. In addition, *I*_2_ is zero, as there is no electromagnetic field distortion. According to Equation (3), *U_O_* is zero at this time. When there are cracks on the surface of the pipeline, the magnetic permeability at the cracks is different from that of the surrounding pipeline. According to Equation (4), as the balanced-field electromagnetic technique sensor moves across the pipeline surface and through the crack, *M*_23_ and *M*_24_ are not equal, causing *I*_3_ and *I*_4_ to be unequal. It breaks the original electromagnetic balance state. *I*_2_ is not zero, and thus, *U_O_* is not zero, which generates a detection signal. In addition, *U_O_* is at its maximum value when the detection coil passes the edges on both sides of the crack. When the detection coil is completely located in the middle of the crack, *I*_3_ and *I*_4_ are equal in size and opposite in direction. At this time, *U_O_* is zero.

**Figure 2:**
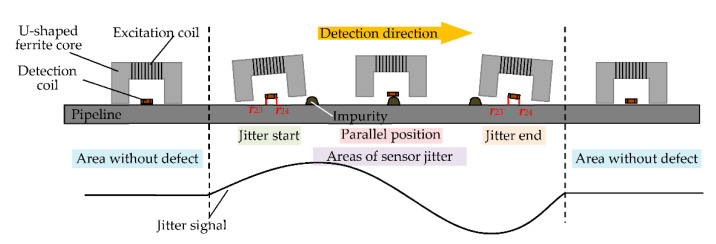
Jitter process of the balanced-field electromagnetic technique sensor.

In the process of detection, the balanced-field electromagnetic technique sensor will not fit well on the surface of the pipeline due to jittering when encountering impurities attached to the surface of the pipeline. The distance between the two legs of the U-shaped ferrite core and the pipeline is not equal, as shown in Figure 2.

It can be seen from Figure 2 that the detection coil is not parallel to the pipeline surface when the sensor is jittering and one of the two legs of the U-shaped ferrite core is closer to the pipeline surface than the other; that is, ***r***_23_ and ***r***_24_ are not equal. According to Equation (4), *M*_23_ and *M*_24_ are not equal, making *I*_3_ and *I*_4_ unequal. Likewise, the electromagnetic field distortion is generated so that *I*_2_ is also not zero, and thus, *U_O_* is also not zero. In this case, *U_O_* is also at its maximum value when the sensor is in the jitter start and jitter end positions. When the sensor is in the parallel position, *U_O_* is zero as ***r***_23_ and ***r***_24_ are again equal, so that *I*_3_ and *I*_4_ are equal in size and in opposite directions. It is worth stating that the lift-off height when the sensor is in the parallel position changes compared to the lift-off height when the pipeline is free of cracks, but no induced voltage is generated in either case due to the self-zero nature of the balanced-field electromagnetic technique [21]. This also shows that no signal will be generated when the balanced-field electromagnetic technique sensor is jittered in the vertical direction. The above shows that the tilt jitter of the sensor is also the reason for the detection signal. The trend of the induced voltage change generated when the sensor jitter occurs is similar to the trend of the induced voltage change generated by the crack, and the jitter signal also generates waveform peaks and valleys similar to those of the crack detection signal. Therefore, it is easy to interfere with the signal discrimination of cracks, which can lead to misjudgments.

### 2.2. Distinguish Method of the Crack Detection Signal

The analysis in Section 2.1. illustrates that the crack detection signal and the jitter signal have similar waveform characteristics, while the angle and depth of the crack can cause changes in signal amplitude [20,35]. Therefore, it is difficult to accurately determine whether the cracks or the jitter of the sensor is the cause of the detection signal by relying on a single signal amplitude change. In addition, it is shown that the detection signal frequency is the same as the excitation frequency from the analysis of the detection voltage mutual inductance model of the balanced-field electromagnetic technique. The crack and jitter are the reasons for the detection signal, and the signals generated by the two have the same frequency, which is equal to the excitation frequency. So, it is not possible to distinguish between the signals generated by cracks and the jitter of the sensor in terms of frequency difference. As another important signal feature besides amplitude and frequency, phase provides a new perspective for crack signal differentiation.

#### 2.2.1. Acquire Signal Characteristics

**Figure 3:**
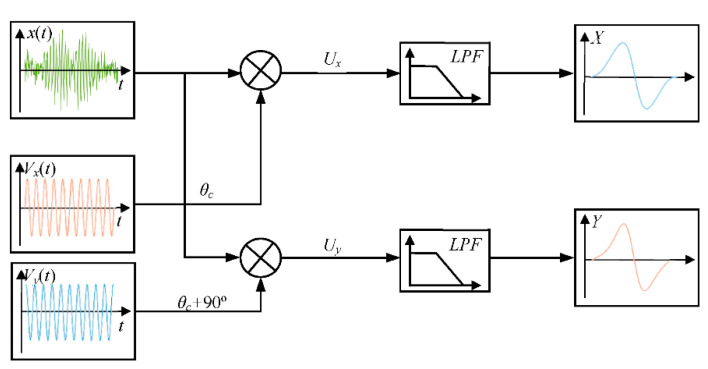
The quadrature demodulation process of the detection signal of balanced-field electromagnetic technique.

The amplitude and phase of the detection signal can be obtained using quadrature demodulation because the balanced-field electromagnetic technique uses alternating current periodic signal excitation. In addition, the detection signal often contains spatial electromagnetic noise that is not at the same frequency as the detection signal due to the complex environment of practical detection. The noise can be filtered out simultaneously by using quadrature demodulation. Figure 3 shows the quadrature demodulation process of the balanced-field electromagnetic technique detection signal.

In Figure 3, *x*(*t*) is the balanced-field electromagnetic technique detection signal with noise, which is expressed as:

(5) $$x(t)=A\sin(2\pi ft+\theta_{0})+\upsilon (t)$$

where *f* is the excitation frequency, υ(t) is the noise signal, $\theta_{0}$ is the detected signal phase, and *A* is the detected signal amplitude.

*V_x_*(*t*) and *V_y_*(*t*) are the in-phase reference signal and the quadrature reference signal, respectively. Their amplitudes are both *B* and their phases are 90° apart, which is expressed as:

(6) $$V_{x}(t)=B\sin(2\pi ft)$$

(7) $$V_{y}(t)=B\cos(2\pi ft)$$

*V_x_*(*t*) and *V_y_*(*t*) are multiplied by the detection signal *x*(*t*), respectively, to obtain two signals *U_x_* and *U_y_*, which are orthogonal to each other.

(8) $$U_{x}=-\frac{AB}{2}[\cos(4\pi ft+\theta_{0})-\cos(\theta_{0})]+\upsilon (t)B\sin(2\pi ft)$$

(9) $$U_{y}=\frac{AB}{2}[\sin(4\pi ft+\theta_{0})-\sin(\theta_{0})]+\upsilon (t)B\sin(2\pi ft)$$

It can be seen that *U_x_* and *U_y_* both contain high-frequency components and noise signals. It can be filtered out by low-pass filtering to obtain the in-phase output component *X* and the quadrature output component *Y* after quadrature demodulation.

(10) $$X=\frac{AB}{2}\cos(\theta_{0})$$

(11) $$Y=\frac{AB}{2}\sin(\theta_{0})$$

It can be seen from Equations (10) and (11) that the original balanced-field electromagnetic technique detection signal with noise is processed into two mutually orthogonal direct current (DC) signals related to the phase of the original signal after quadrature demodulation. From this, the amplitude *A_m_* and phase of the detected signal $\theta_{0}$ can be obtained as:

(12) $$A_{m}=\sqrt{X^{2}+Y^{2}}$$

(13) $$\theta_{0}=\arctan\frac{Y}{X}$$

The above process separates the balanced-field electromagnetic technique detection signal into two components that are orthogonal to each other, and the detection signal amplitude and phase can be obtained by using Equations (10) and (11).

#### 2.2.2. Amplitude-Phase Composite Figure Construction

Although it is not possible to distinguish whether a detection signal is generated by the crack or the jitter of the sensor from the change in amplitude of the signal alone, the amplitude of the detection signal cannot be completely ignored by using the phase of the detection signal as a differentiation method of the detection signal. On the one hand, the detection signal whose amplitude is close to zero does not need to determine whether it is generated by cracks or the jitter of the sensor. On the other hand, when the amplitude of the in-phase component *X* is zero or extremely small and approaching zero, $\frac{Y}{X}$ is close to ±∞ according to Equation (13), so that the phase of the detected signal $\theta_{0}$ is approximately equal to ±90°, and $\theta_{0}$ equals 90° or −90° at random. This causes oscillating noise in the phase observation, which greatly interferes with the phase determination. Therefore, there is a need for a method that can both reflect the amplitude and phase information of the detection signal so as to better realize the discrimination of the crack detection signal.

**Figure 4:**
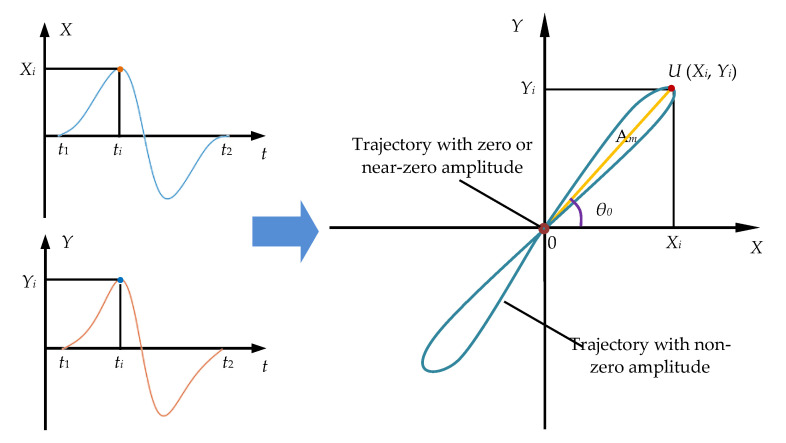
Schematic diagram of the amplitude-phase trajectory of the balanced-field electromagnetic technique.

In Section 2.2.1, the balanced-field electromagnetic technique detection signal is separated by orthogonal demodulation into two components *X* and *Y* that are orthogonal to each other. Equations (10) and (11) are the parametric equations for *X* and *Y*. A rectangular coordinate system in the plane is established with the in-phase component *X* as the horizontal axis and the orthogonal component *Y* as the vertical axis. As shown in Figure 4, the detection signal *U* at any moment *t_i_* can be expressed in the form of coordinates (*X_i_*, *Y_i_*). At the continuous time (*t*_1_, *t*_2_), the detection signal is continuous, and the coordinates corresponding to the detection signal at all moments will form a trajectory in the *XY* plane. According to Equations (12) and (13), the line between the origin and any point on the trajectory represents the amplitude of the detection signal *A_m_*, and the angle between the line representing the amplitude of the detection signal and the *X*-axis represents the phase of the detection signal $\theta_{0}$. In this way, it is included in one figure both the amplitude and the phase of the balanced-field electromagnetic technique detection signal, and the trajectory formed is called an amplitude-phase composite figure.

In Figure 4, the formed amplitude-phase composite figure also presents a more obvious regular pattern when the *X* and *Y* components after quadrature demodulation have obvious peak and valley characteristics. The amplitude-phase composite figure is also different for different amplitudes and phases. In addition, the amplitude-phase composite figure is concentrated around the origin, forming a larger “point” when the detected signal amplitude is zero or close to zero. From an observational perspective, in the amplitude-phase composite figure, the situation where the detection signal amplitude is zero or close to zero and there will be large phase noise is weakened compared to the situation where the *X* and *Y* components have distinct peaks and valleys. This means that the focus is more on the case where the *X* and *Y* components have distinct peaks and valleys, and it ignores the case where the signal amplitude is zero or close to zero, which is meaningless for discriminating between crack and jitter signals. Thus, the detection signal can be discriminated more intuitively and clearly.

## 3. Simulation

### 3.1. Simulation Analysis of Detected Signal Phase

**Figure 5:**
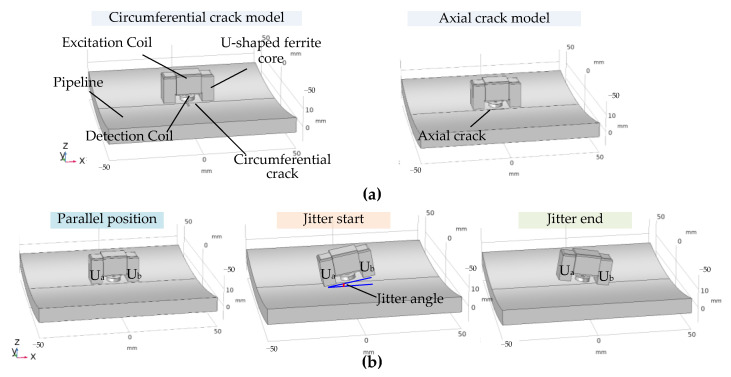
Simulation models for the detection of balanced-field electromagnetic technique: (**a**) Crack detection model; (**b**) Model of sensor jitter.

The amplitude-phase composite figure contains both amplitude and phase information. Since a single change in amplitude cannot distinguish between crack detection signals and jitter signals, it is necessary to ensure that the phase of the crack and jitter detection signals is different so that the amplitude-phase composite figure is distinguishable. In order to further analyze the specific phase of the crack detection signal and the jitter signal, they were established, respectively, in Figure 5a,b as the balanced-field electromagnetic technique cracks detection simulation model and the jitter process simulation model. The sensor dimensions parameters were set with reference to the high-sensitivity balanced-field electromagnetic technique sensor designed by the authors previously [21]. Both the excitation coil and the detection coil were wound with 400 turns, and the material was copper. The relative permeability of the U-shaped ferrite core was 2000, and the conductivity was 0.01 S/m. The thickness of the pipeline was 10 mm, the outer diameter was 457 mm, the length was 200 mm, and the material is X52 steel (electrical conductivity is 0.6 × 10^7^ S/m, relative permeability is 1000). A sinusoidal current with an amplitude of 0.05 A and a frequency of 1000 Hz was applied to the excitation coil to provide an alternating excitation field for the model. This was solved in the Magnetic Fields Interface under the Electromagnetic Fields in the AC/DC module of COMSOL.

**Figure 6:**
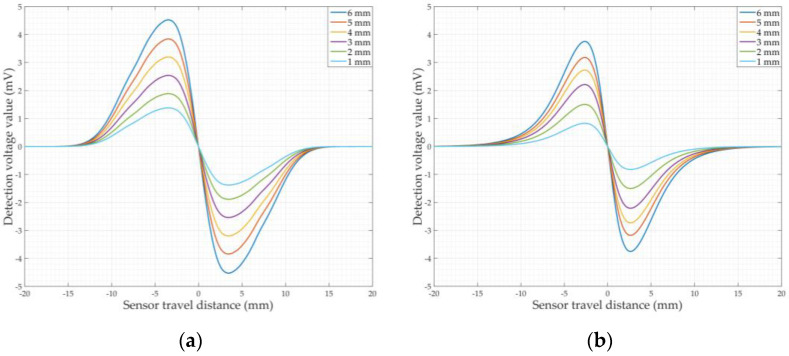

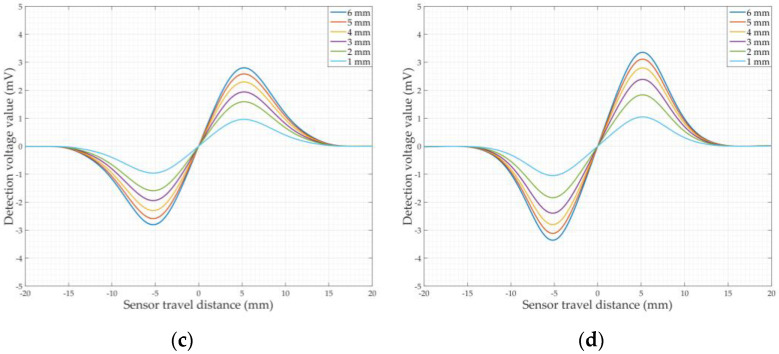
Simulation detection signals of cracks: (**a**) In-phase components of the circumferential crack detection signals; (**b**) Orthogonal components of the circumferential crack detection signals; (**c**) In-phase components of the axial crack detection signals; (**d**) Orthogonal components of the axial crack detection signals.

According to the principle of the balanced-field electromagnetic technique, the detection signals for circumferential cracks (90° to the detection direction) and axial cracks (0° to the detection direction) are the effect of single magnetic flux leakage and a single eddy current field distortion, respectively. The balanced-field electromagnetic technique cracks detection signal at arbitrary angles is generated by the combined effect of the magnetic flux leakage and eddy current field distortion. Therefore, in the crack detection model in Figure 5a, the cracks were set up along the x-direction (axial cracks) and along the y-direction (circumferential cracks) to make the analysis more applicable, respectively. The crack length was 10 mm, the width was 1 mm, and the depth varied from 1 to 6 mm along the z-direction with an interval of 1 mm. In the simulation of crack detection, the geometric center of the crack in the xy-plane was used as the origin, and the starting and ending points of the sensor movement were chosen to be 20 mm to the left and right of the origin along the y-direction. Simulations of 1–6 mm circumferential and axial cracks were carried out separately, and the original detection signals were demodulated orthogonally as in Figure 3 using two reference signals that are orthogonal to each other to obtain the in-phase and orthogonal components of the circumferential and axial cracks, as shown in Figure 6. Figure 6 shows that the peak–valley characteristics of the in-phase and orthogonal components of the circumferential cracks detection signal are a peak followed by a valley, while the peak–valley characteristics of the in-phase and orthogonal components of the axial cracks detection signal are a valley followed by a peak. The peak and valley characteristics of circumferential and axial cracks are opposite. This is because the generation of the circumferential crack signal is dominated by the leakage magnetic flux alone, and the generation of the axial crack signal is dominated by the distortion of the eddy current field alone. This has been demonstrated in the authors’ previous studies [20,35]. The axial cracks detection signal amplitude is smaller than that of the circumferential crack detection signal. As the crack depth increases, the amplitude of the in-phase and orthogonal components of the respective circumferential and axial crack detection signals also increases. This indicates that the depth of the crack affects the amplitude of the signal.

**Figure 7:**
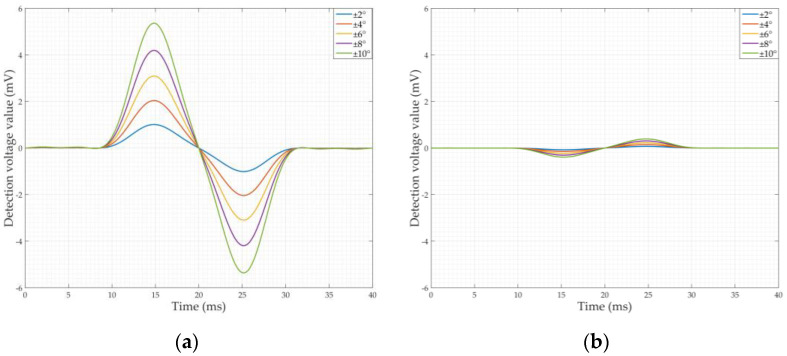
Simulation signals of sensor jitter: (**a**) In-phase components of the jitter signal; (**b**) Orthogonal components of the jitter signal.

In the jitter process model, the pipeline is free of cracks. The simulated sensor jitter process starts in the parallel state and returns to the parallel state after passing through the jitter start state and then the jitter end state, as shown in Figure 5b. Among them, the jitter start is simulated by lifting and falling U_b_ back to the parallel state, and the jitter end is simulated by lifting and falling U_a_ and back to the parallel state. The angle between the plane of the line connecting the two legs of the U-shaped core, U_a_ and U_b_, and the xy plane is the dithering angle (positive for U_b_ lift, negative for U_a_ lift). The jitter angle of ±2°, ±4°, ±6°, ±8°, and ±10° are simulated separately, and the original jitter signal is demodulated orthogonally using two mutually orthogonal reference signals identical to the crack detection signal to obtain the in-phase and orthogonal components of the jitter signal, as shown in Figure 7.

It can be seen from Figure 7 that the waveform characteristics of the signal generated by the sensor jitter are nearly identical to those of the crack detection signal. The amplitude of the in-phase and orthogonal components of the jitter signal increases with the increase in the jitter angle and the jitter signal amplitude is different from that of the crack detection signal. Figure 6 clearly shows that the amplitude of the detection signal is affected by the crack depth; therefore, the above also demonstrates that it is difficult to effectively differentiate between crack and jitter from the signal waveform characteristics and amplitude alone.

**Figure 8:**
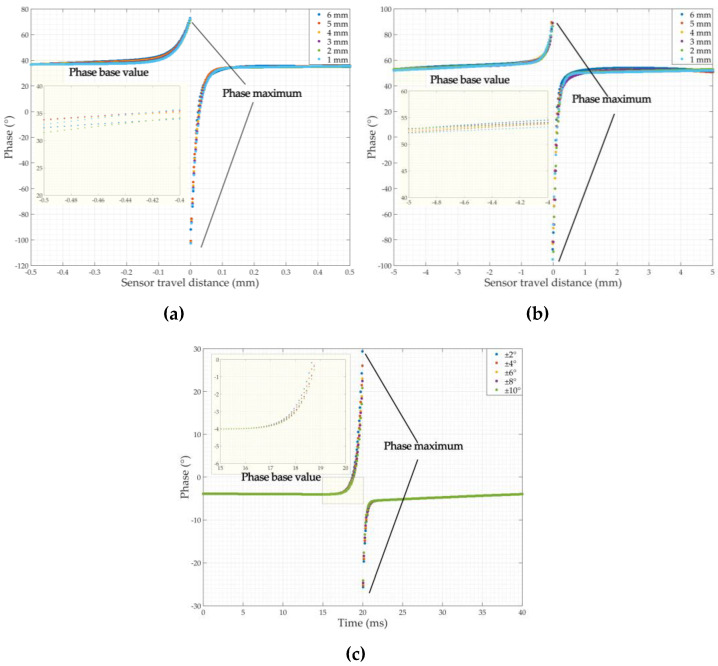
The phase of the simulation signals: (**a**) The phase of the circumferential crack detection signals; (**b**) The phase of the axial crack detection signals; (**c**) The phase of the jitter signals.

The phase of the crack detection signal and the jitter signal were found separately using Equation (13). According to the analysis in Section 2.2.2, there is no need to analyze the cause of the detection signal when the detection signal amplitude is zero (it does not affect the judgment of cracks), and there will also be random phase noise when the in-phase component *X* is zero. Therefore, Figure 8a,b plot the phase of the signal at the crack only, and Figure 8c shows the phase of the jittered signal. According to the analysis in Section 2.1, the position of the phase maximum of the detection signal corresponds to when the detection coil is located right in the middle of the crack or the parallel state of the sensor jitter. At this time, the amplitude of the detection signal is zero. Therefore, only the phase base value of each signal in Figure 8 was analyzed.

It can be seen that the phase base value of the crack detection signal ranges from 35° to 55°. For cracks of the same depth, the phase base value of the detection signal for axial cracks is greater than the phase base value of the detection signal for circumferential cracks; for both circumferential and axial cracks, the phase base value of the detection signal varies in a very small range when the crack depth varies, which indicates that the depth of the crack has a weak effect on the phase. The jitter signal phase base values for different jitter angles are constant, all around −4°, indicating that the jitter angle also has a small effect on the signal phase. In summary, there is a clear distinction between the phase of the crack detection signal and the signal generated by sensor jitter, and the phase is feasible as a characteristic for crack discrimination.

### 3.2. Features of the Amplitude-Phase Composite Figure of the Detected Signal

**Figure 9:**
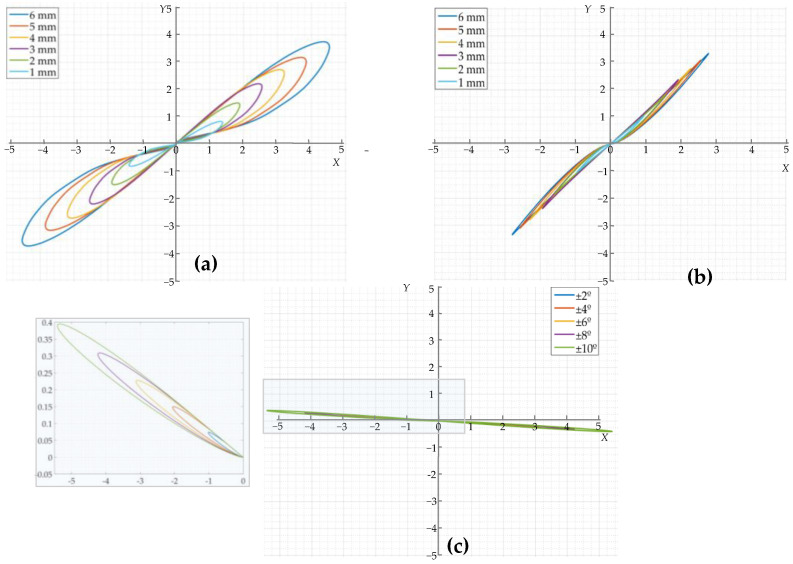
Amplitude-phase composite figure of the detected signals: (**a**) Amplitude-phase composite figure of the circumferential crack detection signals; (**b**) Amplitude-phase composite figure of the axial crack detection signals; (**c**) Amplitude-phase composite figure of the sensor jitter signals.

It was demonstrated that there is a clear distinction between the phase of the crack and sensor jitter signals in Section 3.1. It is necessary to further investigate the characteristics of the amplitude-phase composite figure of the crack and the jitter signal to determine the method of differentiation between the two. According to the study in Section 2.2.2, the respective in-phase components of the crack detection signal and the jitter signal in Section 3.1 were used as the horizontal axis, and their respective orthogonal components were used as the *Y*-axis to plot the amplitude-phase composite figure of the crack detection signal and the amplitude-phase composite figure of the jitter signal, as shown in Figure 9. As can be seen in Figure 9, the amplitude-phase composite figure for both circumferential and axial crack signals are distributed in the first and third quadrants symmetrically about the origin, and the area formed by the amplitude-phase composite figure increases as the depth of the crack increases. The amplitude-phase composite figure for axial cracks has a larger angle to the *X*-axis than the amplitude-phase composite figure for circumferential cracks due to the different phase base values of circumferential cracks and axial cracks. The amplitude-phase composite figure of the jitter signals is distributed in the second and fourth quadrants and is symmetrical about the origin, and the area formed by the amplitude-phase composite figure increases with the increase in jitter angle of the sensor. The angle between the amplitude-phase composite figure and the *X*-axis is extremely small because the amplitude of the orthogonal component of the jitter signal is smaller than that of the in-phase component. This indicates that there is a clear difference between the amplitude-phase composite figure of a crack and the signal generated by sensor jitter, so the distribution of the amplitude-phase composite figure of the detected signal can be used to visually and effectively differentiate between cracks and jitter signals. Furthermore, the amplitude of the simulated signal is almost zero when the pipeline is without defects or when the sensor is not in jitter, so its trajectory in the amplitude-phase composite figure is almost at the origin. This is consistent with the analysis in Figure 4.

## 4. Experiments and Discussion

In order to verify the correctness of the amplitude-phase composite figure characteristics and the effectiveness of the proposed crack detection signal discrimination method, experiments on the crack detection platform with balanced-field electromagnetic technique and practical pipeline engineering applications were carried out, respectively.

### 4.1. Crack Detection Platform Experiments

**Figure 10:**
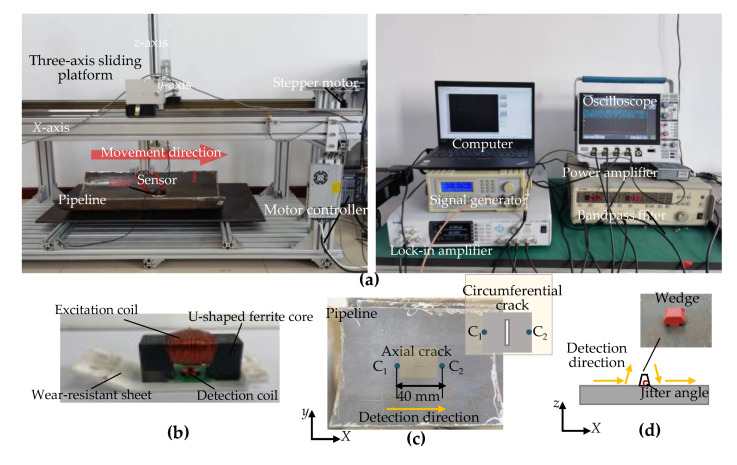
Experimental platform and sensor movement path: (**a**) Detection experimental platform; (**b**) Balance-field electromagnetic technique sensor (**c**) Experimental pipeline physical and movement path of the sensor for the crack detection experiment; (**d**) Movement path of simulated sensor jitter experiment.

In order to prove the correctness of the amplitude-phase composite figure characteristics of the detection signal, the balanced-field electromagnetic technique detection experiment platform was built to carry out pipeline crack detection experiments and simulated sensor jitter experiments, respectively. The balanced-field electromagnetic technique experiment platform uses a three-axis sliding platform as the main body of motion, and a computer sends commands to the motor controller to control the stepper motor to drive the *X*-axis of the three-axis sliding platform to move at a uniform speed. The balanced-field electromagnetic technique sensor is attached to the *z*-axis of the three-axis sliding platform by a flexible bracket and is driven by the three-axis sliding platform to move across the surface of the pipeline. The experiments were carried out in the air, at room temperature, and without strong magnetic interference in the surrounding environment. The signal generator generates a sine wave signal with a frequency of 1 kHz, 100 mV/vpp, which is input to the power amplifier and lock-in amplifier, respectively. The signal generated by the generator is outputted by the power amplifier to a constant current of 50 mA and applied to the excitation coil of the balanced-field electromagnetic technique sensor to provide an alternating excitation field for the experiment. Under the effect of the excitation field, the interaction of the sensor with the pipeline surface is picked up by the detection coil. The detection signal is processed by a band-pass filter (the operating frequency range is 1 Hz–1.59 MHz, and the center frequency is set to 1 kHz) and input to a lock-in amplifier (the operating frequency range is 0.5 Hz–11 MHz. The reference signal frequency is 1 kHz, with a peak-to-peak value of 100 mV, which is consistent with the simulation.). The output signal is displayed and stored by an oscilloscope, as shown in Figure 10a. Among them, the lock-in amplifier completes the orthogonal demodulation process of the original detection signal in Figure 3. The original detection signal was denoised and separated into two mutually orthogonal DC components during the process.

**Figure 11:**
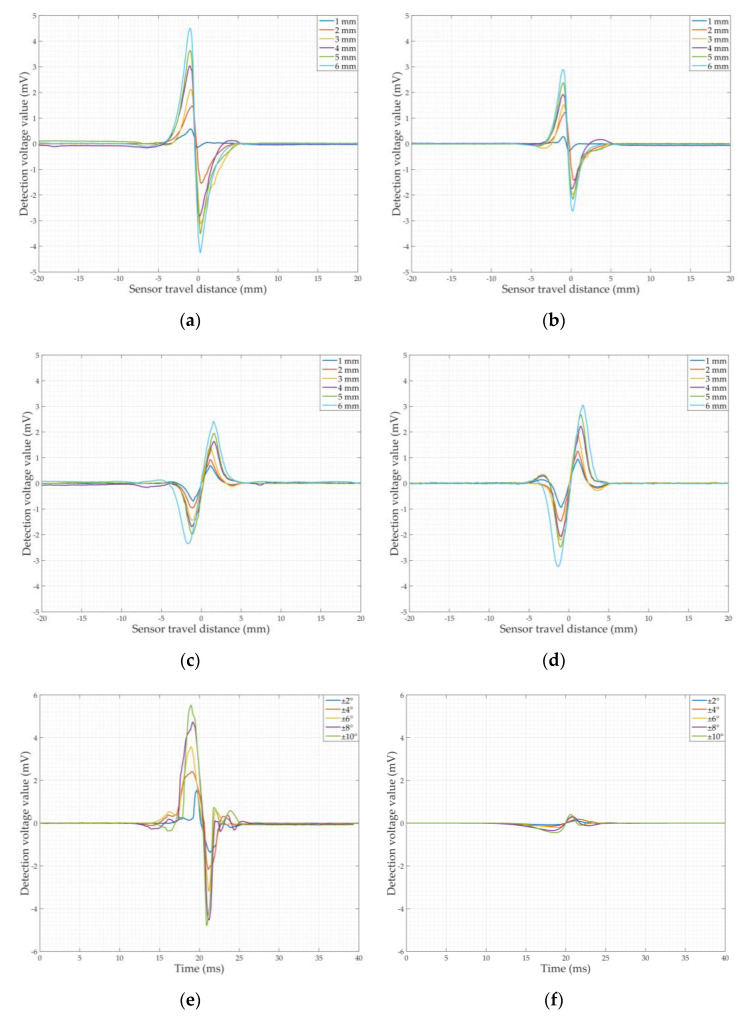
Signals of detection experiments: (**a**) In-phase detection signals of circumferential cracks; (**b**) Orthogonal detection signals of circumferential cracks; (**c**) In-phase detection signals of axial cracks; (**d**) Orthogonal detection signals of axial cracks; (**e**) In-phase components of detection signal generated by sensor jitter; (**f**) Orthogonal components of detection signal generated by sensor jitter.

The experiments were carried out on six sections of the pipeline with EDM cracks in the inner wall and one section without cracks. The diameter and material of all the pipeline sections were identical to the simulation model in Section 3.1 to ensure the consistency of the experimental conditions. The crack length was 10 mm, with the width being 1 mm, and the depth varied from 1 to 6 mm with an interval of 1 mm. The circumferential cracks and axial cracks were detected by changing the direction of the pipeline in relation to the sensor. Figure 10c,d illustrate the movement path of the sensor in the crack detection experiment and the simulated sensor jitter experiment. In the circumferential and axial crack detection experiments, the sensor is moved from point C_1_, 20 mm from the geometric center of the crack in the x-direction, to point C_2_, 20 mm from the geometric center of the crack in the x-direction. The experimental signals for circumferential and axial cracks 1–6 mm deep were obtained as shown in Figure 11a–d. We simulated the detection of sensor jitter angles of ±2°, ±4°, ±6°, ±8° and ±10° by placing non-ferromagnetic insulating plastic wedges of different heights at the geometric center of the inner wall of the pipeline; the jitter detection signals are obtained as shown in Figure 11e,f.

Figure 11 shows that the characteristics of the crack detection signal and the jitter signal of the balanced-field electromagnetic technique are consistent with the simulation analysis, and both have the same peak and valley characteristics. The crack signal amplitude increases with the increase in the crack depth; the amplitude of the jitter signal also increases as the jitter angle increases, and the jitter signal has a larger amplitude than the crack signal. Since the depth of the crack affects the amplitude of the signal, it is also proved in experiments that it is difficult to distinguish from the amplitude of the signal whether it is the crack or the jitter that is the cause of the signal.

**Figure 12:**
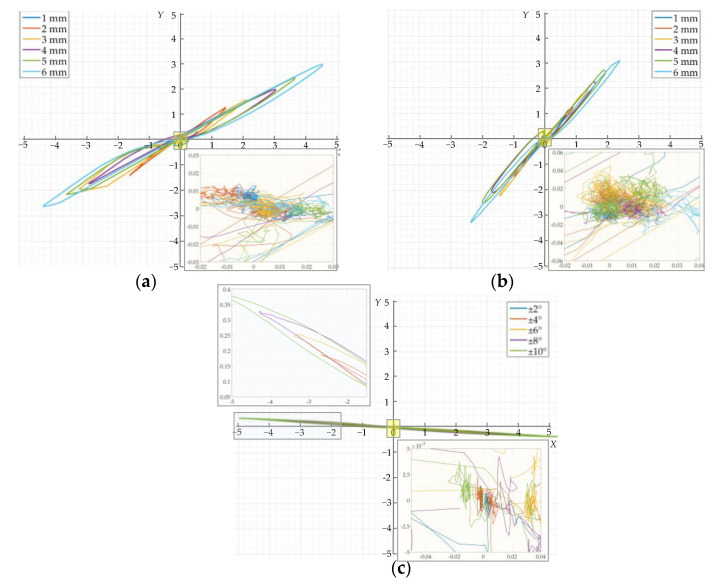
Amplitude-phase composite figure of the experimental signals: (**a**) Amplitude-phase composite figure of the circumferential crack detection signals; (**b**) Amplitude-phase composite figure of the axial crack detection signals; (**c**) Amplitude-phase composite figure of the sensor jitter signals.

The amplitude-phase composite figure of the detection signal was further plotted according to the respective in-phase and orthogonal components of the crack detection signal and the jitter signal in Figure 11, as shown in Figure 12. It can be seen that the amplitude-phase composite figure for circumferential and axial cracks are both symmetrical about the origin in the first and third quadrants and increase in area as the crack depth increases; the amplitude-phase composite figure for axial cracks is closer to the *Y*-axis. The amplitude-phase composite figure of the jitter signal is distributed in the second and fourth quadrants and has a very small angle with the *X*-axis. The above characteristics are consistent with the simulation analysis, which proves the correctness of the proposed amplitude-phase composite figure of cracks and jitter signals in Section 3.2, and the method can be used to distinguish between the crack detection signal and the signal of sensor jitter.

Furthermore, it can be seen from the enlarged section in Figure 12 that it appears haphazard and distributed around the origin in the amplitude-phase composite figure due to the large phase noise when the amplitude of the detection signal is zero or near zero. This is true for both the crack detection signal and the detection signal generated by the sensor jitter. This clustered and haphazard position near the origin is weakened from the point of view of observing amplitude phase two-dimensional figure compared with the case where the in-phase and quadrature components of the detected signal have distinctive peak and valley characteristics. This means that the focus is more on the case where the components of the detection signal have obvious peak–valley characteristics, ignoring the case that the detection signal amplitude is zero or close to zero, which is meaningless for the discrimination of crack and jitter signals in order to make the discrimination of the detected signal more intuitive and clear. This also confirms the correctness of the analysis in Section 2.2.2.

### 4.2. Practical Pipeline Engineering Application

**Figure 13:**
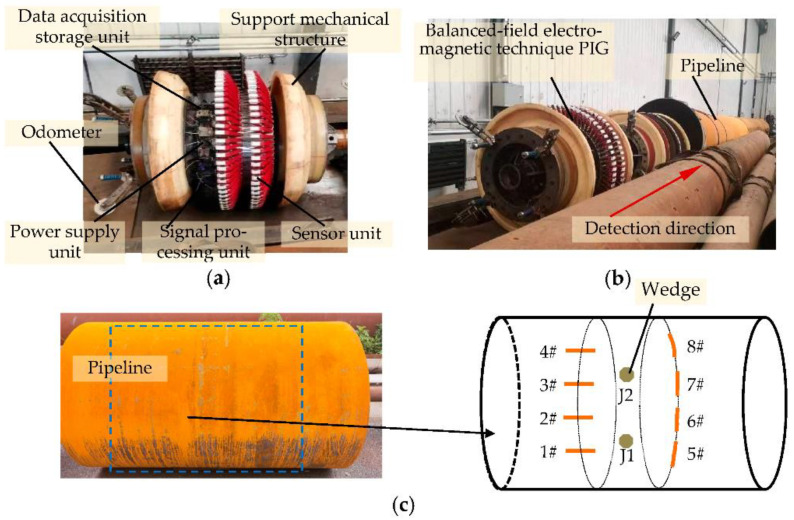
Pipeline field pulling test: (**a**) Balanced-field electromagnetic technique pipeline inspection gauge; (**b**) Pulling test site; (**c**) Schematic diagram of pipeline cracks distribution.

In order to verify the validity and engineering applicability of crack discrimination using the amplitude-phase composite figure of the detection signal, the pipeline pulling test was carried out on the balanced-field electromagnetic technique pipeline inspection gauge (PIG), and the pulling test data were analyzed. Figure 13a shows the balanced-field electromagnetic technique PIG developed by the authors’ research team. It consists of the balanced-field electromagnetic technique sensor unit, the signal processing unit, the support mechanical structure, the data acquisition and storage unit, the power supply unit, and odometers. Among them, the detection core of the balanced-field electromagnetic technique PIG is the sensor unit composed of a number of balanced-field electromagnetic technique sensors that are arranged in two staggered circles and closely adhere to the inner wall of the pipeline. The signal processing unit completes the orthogonal demodulation process of the detection signal and outputs the in-phase and orthogonal components of the detection signal. Then, the data acquisition and storage system records all the detection data, and the odometer records the distance the PIG has traveled. The support mechanical structure allows the PIG to fit closely to the inner wall of the pipeline on the one hand and on the other hand to keep running forward with the help of the pressure difference in the actual running pipeline.

Figure 13c shows the pipeline for the pulling test, which is made of the same material as the simulation model, X52, with an outer diameter of 1219 mm, a wall thickness of 18 mm, and a length of 2 m. Four circumferential cracks and four axial cracks were machined on the inner wall of the pipeline. The circumferential cracks #5–8 are 2–5 mm deep with an interval of 1 mm, all of which are 100 mm long along the circumference of the pipeline and 1 mm long along the axial direction of the pipeline. The axial cracks #1–4 are 2–5 mm deep with an interval of 1 mm, all of which are 1 mm long along the circumference of the pipeline and 100 mm long along the axial direction of the pipeline. In addition, wedges J1 and J2, which simulate the jitter angle of the sensor of ±4° and ±6° in Section 3.1, were fixed to the inner wall of the pipeline at a position axially located at 1.015 m from the start of the pulling test to simulate the jitter process of the sensor.

**Figure 14:**
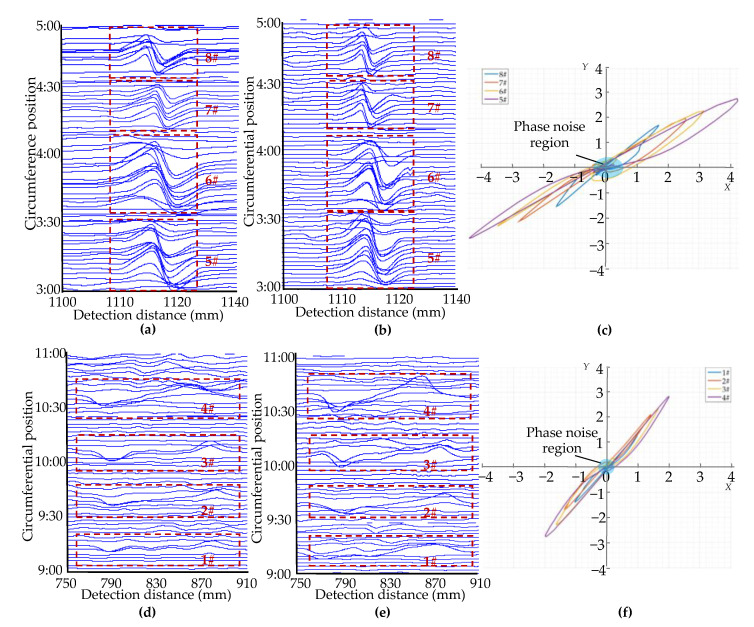
Results of crack pulling test: (**a**) In-phase components of the circumferential crack pulling test signals; (**b**) Orthogonal components of the circumferential crack pulling test signals; (**c**) Amplitude-phase composite figure for circumferential cracks; (**d**) In-phase components of the axial crack pulling test signals; (**e**) Orthogonal components of the axial crack pulling test signals; (**f**) Amplitude-phase composite figure for axial cracks.

**Figure 15:**
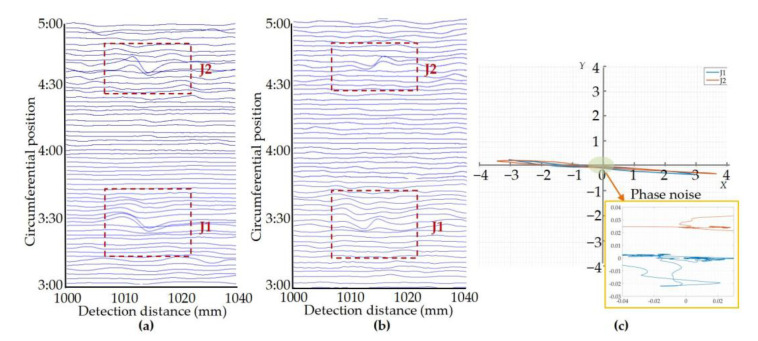
Results of pull-off experiments for sensor jitter: (**a**) In-phase components of the sensor jitter signals; (**b**) Orthogonal components of the sensor jitter signals; (**c**) Amplitude-phase composite figure for the sensor jitter.

A winch was used to pull the balanced-field electromagnetic technique PIG at one end of the pipeline to move and detect in the pipeline. The signal processing system of the balanced-field electromagnetic technique PIG directly outputs the in-phase and orthogonal components of the detection signal after orthogonal demodulation, resulting in the detection results shown in Figure 14 and Figure 15. As the detection signals are the outputs of several sensors, the detection results of each sensor correspond to one signal in-phase component and one signal orthogonal component. In the detection results, the output signals of the different sensors are arranged in the direction of the pipeline circumference, and the circumference of the pipeline is divided into 12 clock positions. To observe the amplitude-phase composite figure clearer, the amplitude-phase composite figure for each crack was plotted by the in-phase component of one with the largest amplitude in the output signals of all the sensors that detect this crack and its corresponding orthogonal component. The amplitude-phase composite figure of the sensor jitter signal was plotted in the same way as the crack. From each of the components of the detection signal, it can be seen that in the form of pulling the balanced-field electromagnetic technique PIG, the detection signal caused by the sensor jitter and the crack detection signal still maintains similar waveform characteristics. The amplitude of the corresponding crack detection signal and the signal generated by the sensor jitter increases as the crack depth increases and the angle of the sensor jitter increases. It can be seen from Figure 14c,f and Figure 15c that the crack amplitude-phase composite figure is still distributed in the first and third quadrants symmetrically about the origin, and the angle between the axial crack amplitude-phase composite figure and the *Y*-axis is smaller than the angle between the circumferential crack amplitude-phase composite figure and the *Y*-axis. The amplitude-phase composite figure of the sensor jitter signals is still distributed in the second and fourth quadrants, with a small angle to the *X*-axis. The amplitude of the detection signal has greater fluctuations around zero at positions where the pipeline is free of cracks and no sensor jitter has occurred, causing a more dramatic phase noise region in the amplitude-phase composite figure. This is because the environment of the pulling test is more complex. The phase noise region remains concentrated around the origin compared to the region where the detection signal has a distinct peak and valley character.

The results of the pulling test again demonstrate the significant difference between the amplitude-phase composite figure characteristics of cracks and sensor jitter, which can be used to distinguish crack detection signal from the signal generated by the sensor jitter. The amplitude-phase composite figure also has a better shielding effect on the phase noise region where the amplitude of the detection signal is zero or close to zero, weakening the observation of the phase noise region where it is meaningless to judge cracks in the pipeline. This feature also makes the use of the amplitude-phase composite figure of the detection signal to identify cracks have better engineering applicability.

### 4.3. Discussions

The results of this study show that the differentiation of crack detection signals from jitter signals in the balanced-field electromagnetic technique by using the amplitude-phase composite figure can be achieved and has a weakening effect on the observation of phase noise. Practical application on the PIG also demonstrates that the method can be used to discriminate engineering inspection data. One of the reasons for the difference in the distribution of the crack and jitter signals in the amplitude-phase composite figure of the detection results is that the peak characteristics of the *X* and *Y* components of the crack signal after quadrature demodulation are identical, whereas the peak characteristics of the *X* and *Y* components of the jitter signal are opposite. This is shown in Figure 11, Figure 14, and Figure 15. On the other hand, it is because the phase base value of the jitter signal is negative, while the phase base value of the crack signal is positive. This is also the reason for the extremely small angle between the amplitude-phase composite figure of the jitter signal and the *X*-axis. In addition, the peak and valley values of the signals in Figure 11e,f are not perfectly symmetrical because the crack detection experimental platform cannot make the sensor pass through the wedge at exactly the same angle during the process of driving the sensor movement; i.e., the jitter start angle and jitter end angle of the experiment are not exactly the same. However, this does not affect the apparent difference in the distribution of the crack and jitter signals on the amplitude-phase composite figure, and this method can still be used to distinguish the two.

Since the crack detection signal of the balanced-field electromagnetic technique and the jitter signal are similar in waveform and have the same frequency, the commonly used crack image identification methods and frequency difference identification methods are not suitable for the signal distinction of the balanced-field electromagnetic technique. There is also no published research content on the detection of signals of the balanced-field electromagnetic technique currently. This paper provides a feasible method for the differentiation of crack and jitter signals of the balanced-field electromagnetic technique. However, the method also has certain shortcomings in practical applications. For example, the signal on the PIG is composed of multiple sensors, and the amplitude of the detection signal will be affected by the surrounding sensors due to the AC excitation. Therefore, there is a certain deviation in the range of amplitude variation of the detected signal from the simulation and the platform experiment. This can be solved by adjusting the circumferential spacing of the PIG’s sensor arrangement to minimize the influence of the excitation field between channels.

In addition, the distribution characteristics of the amplitude-phase composite figure and the phase base values of jitter and cracks presented in this study are applicable to X52 steel pipelines. This material is a typical material for long-distance oil and gas pipelines according to the standard API 5 L of the American Petroleum Institute and the standard ASTM A106 of the American Society for Testing Materials. As different grades of carbon steel are used for different pressure pipelines, future research will be carried out on the distinction between jitter and crack for the pipeline of more materials so that the proposed method can be more perfect in the application of actual long-distance oil and gas pipelines. Further research will also be carried out on the method of eliminating the interference signal generated by the sensor tilt jitter in the future, so as to reduce the misjudgment of the detection signal at the source and improve the engineering practicality of the balanced electromagnetic technology.

## 5. Conclusions

Due to the inevitable tilt jitter of the balanced-field electromagnetic technique sensor during pipeline in-line inspection, an interference signal similar to the waveform of the crack detection signal will be generated. To address this problem, this paper proposes a method of differentiating the crack detection signal from the signal generated by the sensor jitter by using an amplitude-phase composite figure. The principle of the detection signal generated by crack and sensor jitter has been analyzed by the balanced-field electromagnetic technique mutual inductance model, and the amplitude-phase composite figure has been constructed by the detected signal components after orthogonal demodulation. The amplitude and phase of the crack detection signal and the sensor jitter signal have been analyzed by finite element simulation, and the characteristics of the amplitude-phase composite figure have been determined. The correctness of the theoretical analysis and the effectiveness of the proposed differentiation method have been verified by platform experiments and dynamic pulling tests. The research results show the following:

(1) The crack detection signal and the signal generated by the sensor jitter are of the same frequency and have similar waveform characteristics. The increase in crack depth and the increase in the angle of the sensor jitter will increase the amplitude of the detection signal so that it is not possible to distinguish between crack and sensor jitter by the amplitude of the signal alone.
(2) There is a significant difference between the phase base value of the crack detection signal and the phase base value of the signal generated by the sensor jitter. For X52 steel pipes, the phase base value of the crack detection signal is between 35° and 55° when the crack depth is certain, and the phase base value of the axial crack detection signal is larger than that of the circumferential crack detection signal; the effect of crack depth on the phase of the detection signal is weaker. The phase base values of the different jitter angle signals of the sensor are all −4°. Since phase noise will be generated when the detection signal amplitude is zero or close to zero, it is not possible to distinguish between crack and sensor jitter by the phase of the signal alone.
(3) The amplitude-phase composite figure characterizes both the amplitude and the phase of the detected signal. The amplitude-phase composite figure of the crack detection signal is distributed symmetrically about the origin in the first and third quadrants, and the amplitude-phase composite figure of the axial cracks is closer to the *Y*-axis than the amplitude-phase composite figure of the circumferential cracks. The amplitude-phase composite figure of the signal is distributed in the second and fourth quadrants and has a very small angle to the *X*-axis. With the increase in crack depth and sensor jitter angle, the area of the amplitude-phase composite figure will increase. The difference in the characteristics of the amplitude-phase composite figure between the crack and the sensor jitter signal can be used to distinguish between the two.
(4) From the observation point of view, the use of the amplitude-phase composite figure can effectively shield the phase noise region in the detection signal of the balanced electromagnetic technology and focus more on the position of the detection signal with obvious peak and valley characteristics, which is convenient to distinguish the crack and the sensor jitter signal intuitively and clearly.

## Data Availability

Not applicable.
